# Astaxanthin limits atherosclerosis and dysmetabolism in mice by attenuating inflammatory cell recruitment and signaling

**DOI:** 10.1371/journal.pone.0334410

**Published:** 2025-10-31

**Authors:** Nathaly Anto-Michel, Clemens Diwoky, Katharina Pfeil, Heinrich Mächler, Andreas Zirlik

**Affiliations:** 1 Department of Cardiology, University Heart Center Graz, Medical University of Graz, Graz, Austria; 2 Institute of Molecular Biosciences, University of Graz, Graz, Austria; 3 Department of Cardiac Surgery, University Heart Center Graz, Medical University of Graz, Graz, Austria; Hong Kong Baptist University, HONG KONG

## Abstract

**Introduction:**

Astaxanthin (ASX) has demonstrated various cardioprotective effects, including reductions in body weight, adipose tissue mass, hypertension, myocardial infarct size, and oxidative stress markers. Despite these findings, the underlying mechanisms remain unclear. This study examines the role of ASX in murine atherosclerosis and metabolic derangements induced by atherogenic diet, aiming to gain a deeper understanding of its biological effects and potential therapeutic applications.

**Methods:**

Ldlr-/- mice were fed a high-fat, high-cholesterol diet (HCD) for 16 weeks, receiving 70 mg/kg ASX or vehicle every other day. A week before the study ended, glucose and insulin tolerance tests were performed. Plaque size in the aorta was analyzed via histology (Oil-red-O and Masson’s trichrome). Flow cytometry assessed immune cells from blood, aorta, adipose tissue, and cytokines in plasma. Additional mice underwent intravital microscopy for further investigation.

**Results:**

The overall body weight of animals treated with ASX or vehicle did not differ. ASX-treated mice showed a reduced abundance of peripheral monocytes by 34%, lower numbers of leukocytes in adipose tissue depots, and improved glucose metabolism and insulin sensitivity compared with animals receiving vehicle. White adipose mass decreased while brown adipose and muscle mass increased in mice treated with ASX. Atherosclerotic lesions of Ldlr^-/-^ mice receiving ASX were significantly smaller and contained fewer lipids (3.3 vs 2.6 x 10^5^ µm2) and M1 macrophages (0.97 vs 0.42x10^3^) but increased collagen, in line with a more stable plaque phenotype. Mechanistic experiments revealed that ASX attenuated leukocyte recruitment (43% ± 1.87) to the vessel wall in intravital microscopy and dampened inflammatory signaling through Mitogen-activated protein kinases.

**Conclusion:**

ASX treatment reduces experimental atherosclerosis and blunts metabolic syndrome features in mice. This effect is linked to reduced leukocyte recruitment and systemic/local inflammation. The findings support ASX’s potential in treating atherosclerosis and metabolic diseases, offering new mechanistic insights and ultimately warrant the rigorous clinical evaluation of such putative effects.

## Introduction

Atherosclerosis is a chronic inflammatory disease driven by immune and inflammatory responses at all stages, from onset to complication. Despite advances in cardiovascular treatments, it remains a leading global cause of death [1,2]. Recent research highlights the role of immune cells, opening doors to therapies like antigen-specific immunomodulation and vaccines [3].

Obesity, especially visceral fat from poor lifestyle habits, significantly raises the risk of atherosclerosis and related events such as heart attack and stroke [4]. When combined with hypertension, dyslipidemia, and insulin resistance, it forms metabolic syndrome—a major cardiovascular threat now reaching epidemic levels worldwide [5].

Interestingly, obesity and, more so metabolic syndrome come with a systemic increase in inflammatory markers known to associate with cardiovascular events and outcome [6]. Obese visceral adipose tissue represents a highly active endocrine organ and a major source of such cytokines (pro-inflammatory marker C-reactive protein, tumor necrosis factor α and interleukin-6) and adipokines (resistin, visfatin, adiponectin and leptin), likely promoting deleterious crosstalk between pathologically activated components of the immune system and the vascular endothelium. These phenomena ultimately result in the development of atherosclerotic plaque [7]. In fact, upon onset of obesity [8,9], classically activated macrophages and CD8^+^ T-cells infiltrate adipose tissue in ways very similar to their infiltration of nascent atherosclerotic plaques, rendering obesity an inflammatory disease with similar properties as atherosclerosis [10].

**Fig 1:**
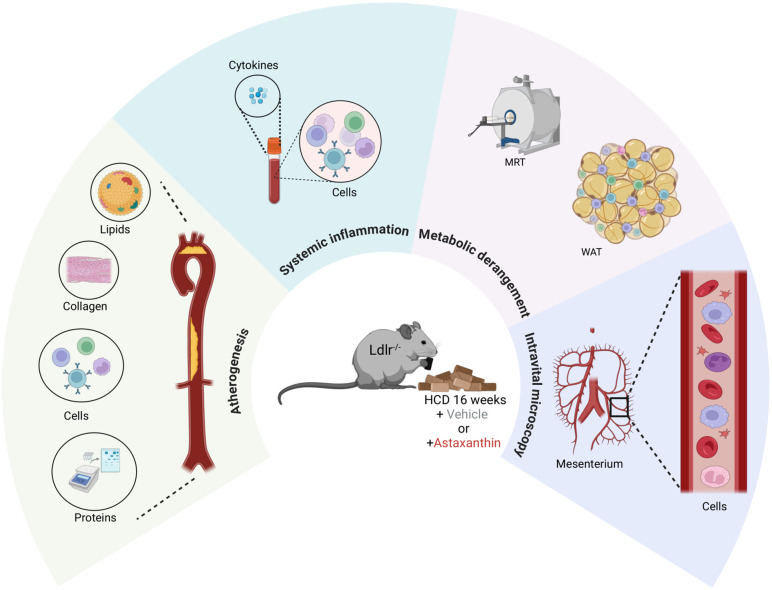
Scheme of the experimental design. Atherogenesis, metabolic parameters, inflammation and molecular pathways with the help of histology, flow cytometry, MRT and intravital microscopy were assessed Ldmlr^−/−^ mice on HCD received ASX or vehicle every second day for 16 weeks.

Astaxanthin (ASX) is a fat-soluble xanthophyll carotenoid synthesized from seaweed, such as Chlamydomona, naked algae, Cynaobacteria or Heamaytococcus pluvalis [11]. The latter presents a higher ASX extraction yield (1.5%−3%) and might be the best biological source of ASX [12]. Therefore, the best product choice is the indoor system employing LED light from Biolife, Austria. Here, the substrates of the freshwater algae are growing; they are reddened, separated, and dried. In the last ten years, ASX presented considerable antioxidant properties. Various preclinical studies associated ASX exposure with favourable cardiovascular effects, such as reduction of inflammatory biomarkers, markers of oxidative stress and lipid peroxidation, fat accumulation, blood pressure, and infarct size after experimental myocardial infarction [13–16]. Three promising placebo control clinical trials described that supplementation with ASX (from 8 to 20 mg/day) improves LDL, HDL, and triglyceride levels, decreases oxidation of fatty acids, and reduces biomarkers of oxidative stress, such as malondialdehyde and isoprostanes [15,17,18]. In male Swiss albino mice fed a high-fat-high-fructose diet, astaxanthin supplementation (6 mg/kg/day) for 60 days improved insulin sensitivity by enhancing insulin receptor signaling and reducing oxidative stress and inflammation in skeletal muscle and adipose tissue [19]. In a rat model of peritoneal fibrosis, oral administration of astaxanthin attenuated the infiltration of monocyte chemoattractant protein-1 (MCP-1)-positive cells. It suppressed MCP-1 mRNA expression, indicating its potential in preventing fibrosis-related complications [20]. Supplementation with astaxanthin-rich extract for 30 weeks resulted in significant reductions in body weight, serum and liver lipid accumulation, and aortic atherosclerotic lesions. Additionally, ASX improved retinal health and modulated gut microbiota, indicating systemic benefits beyond lipid regulation [21]. It has also been reported that ASX supplementation promoted reverse cholesterol transport (RCT), a process that facilitates the removal of excess cholesterol from macrophages and reduces aortic cholesterol content [22]. These studies highlight that ASX can affect the development of atherosclerosis. However, the exact mechanism of ASX in atherosclerosis development is unknown. Here, we investigate the impact of ASX on experimental atherosclerosis in Ldlr^−/−^ mice (Fig 1).

## Materials and methods

### Animals and treatment

Male and female Ldlr knockout mice 8-week-old were used and housed in conventional cages under standard conditions and *ab libitum* access to water and High cholesterol diet (HCD) containing 1.25% cholesterol and 17% cocoa butter (Sniff Spezialdiäten GmbH, D1208). Animals received Astaxanthin (70 mg/kg) or vehicle (sunflower oil) every other day via gavage (*n* = 15 per group). Mice were on HCD and treatment for 16 weeks. At the end of the study animals were harvested and histologically analyzed as described previously [23,24].

The mice were purchased from Jackson Laboratory (B6.129S7-Ldlrtm1Her/J). All mouse experiments were performed in accordance with the Guide for the Care and Use of Laboratory Animals published by the US National Institutes of Health and were performed after securing approval from the Federal Ministry of Education, Science, and Research, Austria. (TVG: 2021-0.009.980)

The method of euthanasia used was Carbon dioxide (CO2) inhalation, and depending on the experiment, analgesics (ketamine and xylazine) were used to eliminate/alleviate suffering.

### Oil Red O staining

Frozen sections were fixed with 10% neutral buffered formalin for 10 min at room temperature. Sections were incubated with propylene glycol for 2 min, then with pre-heated Oil Red O staining solution (American MasterTech, STOROPT) at 60 °C for an additional 8 min. Sections were placed in 85% propylene glycol for 1 min and counterstained with modified Mayer’s hematoxylin (American MasterTech, HXMMHLT) for 1 min, rinsed with water, and then mounted with glycerine jelly (Fisher Scientific, NC0301797).

### Masson’s trichrome staining

Aortic roots were cut into 5-µm sections and stained by Masson’s trichrome stain to detect collagen deposits. Instructions were followed as indicated by Sigma Aldrich (HT15).

### Adipose tissue arrangement

Body composition was determined using a 7 Tesla (Biospec 70/20, Bruker, Ettlingen, Germany). Anesthesia was induced by placing the mice for 3–4 min in a chamber of 4% isoflurane in O2. During magnetic resonance imaging (MRI), the anesthetic level was maintained by 1.8–2.0% isoflurane in O2 (1 L/min) to keep the respiration rate between 80 and 100 bpm. Mice breathed freely, and animal body temperature was maintained by a water-heated animal bed. Mice were scanned in supine position. A balloon pressure sensor (SA Instruments Inc, NY, USA) was placed on the abdomen for respiratory gating. For adipose tissue segmentation, a T2-weighted respiratory gated 2D fast spin echo sequence was used with the following parameters: TR/TE = 2200/28.8 ms, turbo factor = 13, slice thickness = 500 µm, in-plane resolution = 156 × 156 µm, FOV = 40 × 40 mm, 30 slices. The ingWAT, PVAT, and visWAT were manually segmented in ITK-Snap [25]. For quantitative lipid mapping, a proton density fat fraction measurement was carried out [26] employing a respiratory-gated multi-echo gradient echo sequence with the parameters: TR = 700 ms, alpha = 20°, 8 monopolar echoes with TE = 1.6/2.8/3.9/5/6.2/7.3/8.4/9.6 ms, slice thickness = 500 µm, in-plane resolution = 219 × 219 µm, FOV = 35 × 35 mm, 55 slices. Proton density fat fraction was fitted from the multi-echo data using a variational approach, including B0-field correction [27].

### Intraperitoneal glucose tolerance testing (GTT) and insulin tolerance testing (ITT)

Glucose and insulin tolerance testing was performed after 16 weeks of HCD as described previously [28]. In brief, mice were fasted 8 hours before the test. For GTT, mice received an intraperitoneal glucose injection (1g/kg body weight). For ITT, mice were injected with human insulin intraperitoneally (0.5 U/kg, Actrapid Insulin, Novo Nordisk, Denmark). Blood samples were collected at 0, 15, 30, 45, 60, 90, and 120 minutes after the glucose or insulin administration, and plasma glucose concentration was measured with an AccuChek GO (Roche Diagnostics, Switzerland). As indicated, area-under-the-curve analysis (glucose concentration in mg/dl over time in minutes) was calculated per animal.

### Isolation of stromal vascular fraction (SVF)

Murine visceral fat pads were minced and digested with collagenase I (0.5 mg/ml) for 30 minutes at 37°C. Digested tissue was filtered through a cell strainer (100 µm). The adipocyte fraction was collected from the floating top layer, and the pellet (SVF) was collected for further FACS analysis.

### Flow cytometry

FACS analysis was performed as described previously [29]. In brief, cells were washed in PBS and stained with viability dye for 30 min on ice. Cells were washed with FACS buffer (PBS^−/−^/1% FCS) and incubated with the indicated antibodies (all from Biolegend, 1:200) before quantification on a flow cytometer (CytoFlex Beckman). Leukocyte populations were identified as CD45 + viability dye- cells and upon cell surface expression of the indicated antigens (S1 Table): T-helper cells (CD3+ CD4+ CD8−), cytotoxic T cells (CD3+ CD4− CD8+), T-regulatory cells (CD4+ CD25+ FoxP3+), B cells (CD19+) or dendritic cells (F4/80− CD11c+). Adipose tissue macrophages were identified as CD45+ CD11b+ CD3− CD19− F4/80+ or M1 macrophages + CD11c+.

### Plasma sample analysis

Plasma levels of FFAs were measured by a commercial enzymatic method using 3-methyl-N-ethyl-N- (β-hydroxyethyl)-aniline (MEHA) as a violet color agent according to the manufacturer’s protocols (LabAssay NEFA, USA). An enzymaticcolorimetric test determined triglyceride and total cholesterol levels (DiaSys, Germany).

### Inflammatory markers in plasma

Levels of Interleukin-6 (IL-6), Interleukin-10 (IL-10), Monocyte Chemoattractant Protein-1 (MCP-1), Interferon-γ (IFN-γ), Tumor Necrosis Factor (TNF), and Interleukin-12p70 (IL-12p70) were determined by bead array (BD™ Cytometric Bead Array (CBA)).

### Gene expression

Freshly collected abdominal aortas were stored in RNAlater (Qiagen, Germany). RNA was extracted by TrizolLS (Invitrogen, USA) and glycogen as a co-precipitator (Roche, Switzerland). Homogenization was performed using a rotor-stator dispergator (IKA, Germany). 1 µg of total RNA was transcribed into cDNA using the iScript Synthesis Kit (Bio-Rad, Germany). Subsequent quantitative PCR was performed using the SYBR green Master Mix (ThermoFischer, USA) detection format. α-Actin and GAPDH served as control as indicated (S2 Table).

### Intravital microscopy

Intra-vital microscopy was performed as previously described [30]. In brief, mice on WD and ASX or vehicle per 10 days before surgery received intraperitoneal injections of Murine TNFα, 200ng (R&D systems), and surgery started four h later. Briefly, mice were anesthetized by intraperitoneal injection of ketamine hydrochloride (100 mg/kg; Essex, USA) and xylazin (5 mg/kg; Bayer, Germany). The mesentery was exteriorized and placed under an upright intravital microscope (AxioVision, Carl Zeiss, Germany). Videos of rolling and adhering in mesenteric venules were taken after retro-orbital injection of rhodamine (0,025 mg/kg; ~60 μL of 1 mg/mL). Rolling leukocyte flux was defined as the number of leukocytes moving at a velocity less than erythrocytes. Adherent leukocytes were defined as cells that remained stationary for at least 30 s.

### Western blot

Aorta tissue samples were lysed, separated by SDS-PAGE under reducing conditions, blotted to polyvinylidene difluoride membranes, and stained against the indicated antibodies, total p38 MAPK (Cell Signaling, 9212S, 1:1000), phosphorylated p38 MAPK (Cell Signaling, 9211S, 1:1000), followed by a visualization with Clarity Western ECL Substrate (Biorad, 170–5061). For quantification of protein expression, the density of the specific targets was normalized to GAPDH (Clone 14C10, Cell Signaling 2118S, 1:1000) and expressed as the ratio of phosphorylated protein/total protein.

### Statistical analysis

Data are presented as mean ± standard error of the mean (SEM), and p-values less than 0.05 were considered statistically significant. Statistical testing employed a 2-sided, unpaired Student’s T-test between the groups for normal distributed variables and a Mann-Whitney Test for non-normal distributed variables. Differences across three or more groups were tested with ANOVA with Turkey’s multiple-comparison test or multiple T-tests with Holm-Sidak corrections for multiple comparisons. For time courses of two groups, repeated measures, 2-way ANOVA was tested.

## Results

### Astaxanthin treatment results in smaller atherosclerotic lesions showing features associated with plaque stability

**Fig 2:**
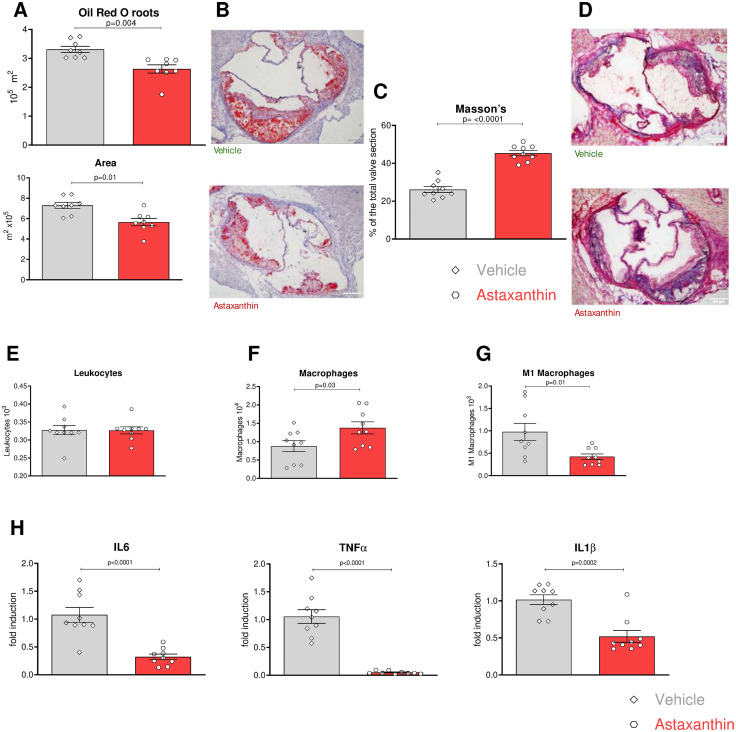
Atheroprotective effect of Astaxanthin. **(A)** Quantification of lipid content and atherosclerotic plaque area. **(B)** Representative picture of the aortic root sections stained with Oil-Red-O (ORO) and hematoxylin. **(C)** Sections of the aortic root were analyzed for collagen content; positive staining is displayed as mean±SEM duplicates (N = 9 per group, 5 males, 4 females). **(D)** Masson’s Trichrome staining. **(E, G)** Total Leukocytes, macrophages and M1 pro-inflammatory population identified in the digested aorta (n = 9 per group, 5 males, 4 females) by flow cytometry. **(H)** Gene expression of IL6, TNFα, and IL1β on qPCR from whole abdominal aorta. An unpaired T-test assessed (A, C, E-G) Significance.

To investigate the role of ASX in murine atherogenesis, Ldlr^−/−^ mice consumed a high cholesterol diet (HCD) supplemented with ASX or vehicle (sunflower oil) for 16 weeks, starting at 8 weeks of age. Animals treated with ASX (5.65 × 10^5^ µm^2^ ± 0.36) exhibited significantly smaller atherosclerotic plaque areas at 16 weeks than animals receiving vehicle (7.29 × 10^5^ µm^2 ^± 0.36, N = 18, P = 0.01). Histologic analysis of plaque composition revealed that atherosclerotic lesions from ASX-treated animals had reduced lipid content as measured by Oil-red—O (3.3 × 10^5^/µm^2^ ± 0.10 in vehicle vs. 2.6 × 10^5^/µm^2^ ± 0.14 in ASX, N = 18, P = 0.004, Fig 2A and 2B). In contrast, while total leukocyte numbers did not differ between the groups (Fig 2E), overall macrophage infiltration increased in the plaques of ASX-treated mice; however, the M-1 subtype, known to be pro-atherogenic, was significantly less abundant (Fig 2F and 2G) in the ASX group (0.97 × 10^3 ^± 0.18 in vehicle vs 0.42 x10^3 ^± 0.06 in ASX, N = 18, P = 0.03). Other leukocyte subpopulations, such as granulocytes, T helper, and T cytotoxic cells, showed no changes (S1 Fig A-C). Given that M1 macrophages release pro-inflammatory cytokines during inflammation, we examined the gene expression of IL-6, TNFα, and IL-1β in the aorta. Consistent with the observed reduction in inflammatory M1-polarized macrophages, the overall inflammatory burden of plaques from ASX-treated animals was significantly lower, as indicated by very low expression levels of IL-6, TNFα, and IL-1β (Fig 2I). Further morphological analysis of the roots using Mason’s trichrome staining also revealed increased collagen content (26.14% ± 1.54) compared to the control group (45.34% ± 1.37, N = 18, P = 0.001) (Fig 2C and 2D). Overall, this extensive phenotypic analysis suggests that the smaller atherosclerotic lesions seen in ASX-treated animals are less inflamed due to a decrease in the pro-inflammatory M1 phenotype of macrophages, which correlates with reduced production of pro-inflammatory cytokines and indicates signs associated with increased plaque stability [31–33].

### Animals treated with Astaxanthin show reduced numbers of inflammatory monocytes and attenuated levels of pro-inflammatory cytokines

**Fig 3:**
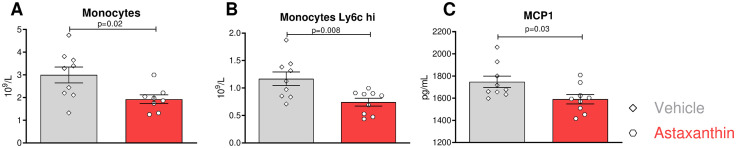
Astaxanthin’s effects on circulating monocytes. **(A)** Monocyte subsets were identified using flow cytometry in peripheral blood following the lysis of erythrocytes. **(B)** Surface expression of Ly6c on peripheral monocytes was analyzed by flow cytometry. **(C)** Plasma levels of MCP1 were measured with a CBA kit. **(A-C)** Significance was evaluated with an unpaired T-test. N = 9/8 (5 males, 4 females vs 4 males and 4 females).

Flow cytometry analysis of the blood showed that the fraction of circulating monocytes analyzed by expression of CD45^+^/CD11b^+^/CD115^+^/Ly6G^−^ was reduced from 2.9 × 10^9^/L ± 0.35 in the vehicle group to 1.9 × 10^9^/L ± 0.19 in the ASX group (N = 18, P = 0.02, Fig 3A-3C). At the same time, other myeloid subpopulations were unaffected by ASX treatment (S2 Fig A, B). Notably, the numbers of inflammatory monocytes expressing high levels of Ly6C were reduced by 36% (Fig 3B), a cell fraction known to propagate atherosclerosis and its sequelae [34,35].

The number of circulating B and T cells and their different subsets did not show significant differences between the control and treated groups (S2 Fig D-H). However, analysis of inflammatory cytokines in the plasma by cytokine bead array revealed significantly decreased levels of pro-inflammatory MCP1 cytokine in the ASX group (1591pg/mL ± 42.20) compared to the control group (1748pg/mL ± 51.44, N = 18, P = 0.03, Fig 3C), whereas concentrations of TNFα, IFNγ, IL10, IL-6, and IL-12 cytokines did not differ between the two groups (S2 Fig I-M). These data strongly suggest that ASX also enfolds less pro-inflammatory immune profile in the periphery.

### Astaxanthin limits inflammatory cell recruitment to the vessel wall and reduces MAP-kinase activation

**Fig 4:**
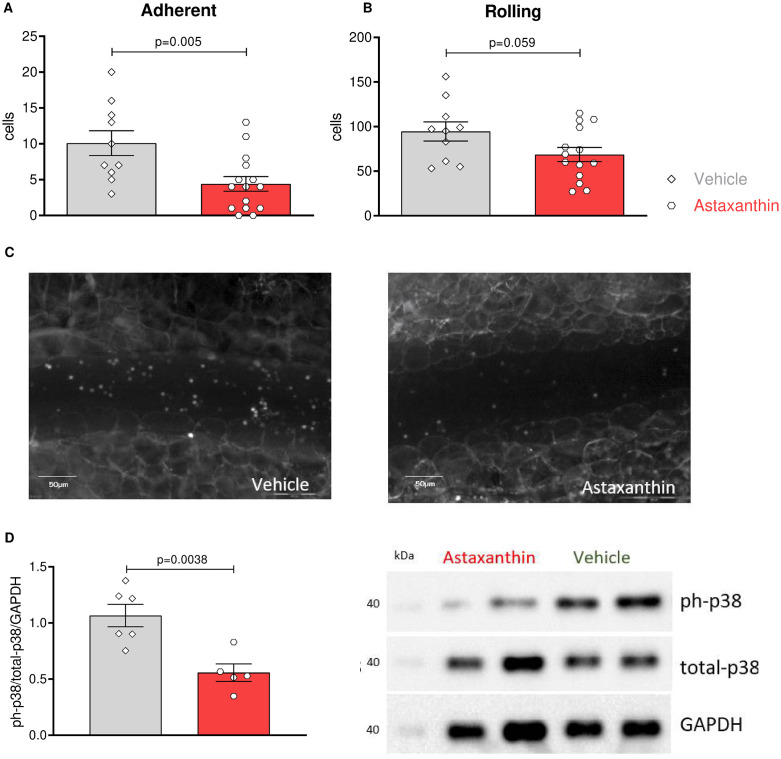
Treatment with Astaxanthin inhibits leukocyte infiltration and MAP kinase activation. Leukocyte recruitment was observed via intravital microscopy following 4 hours of TNFα stimulation. **(A)** Quantification of adherent and **(B)** rolling leukocytes was performed. Error bars represent mean ± SEM. Statistical significance was determined using an unpaired T-test between the indicated groups. N = 10/15. **(C)** Representative images of intravital microscopy. **(D)** Protein was isolated from the whole aorta, and both total and phosphorylated p38 were visualized by western blot; the ratio of phosphorylated fractions was calculated and normalized to the housekeeping GAPDH. Values were expressed as relative arbitrary units (AU) normalized to the signal from the animals in the control group receiving only the vehicle. N = 6 per group (3 males and 3 females).

To explore mechanisms explaining the reduced content of inflammatory macrophages in the atherosclerotic plaques of ASX-treated animals, we tested whether ASX may affect the rolling and adhesion of inflammatory cells, crucial steps in the recruitment of inflammatory cells to sites of inflammation. Therefore, mice were subjected to intravital microscopy in the presence of Astaxanthin or vehicle 1 day before challenging them with systemic injection of TNFα for four hrs. Fewer leukocytes rolled (94.5 cells ± 10.68 vs 68.64 cells ± 7.95, N = 20, P = 0.05) and adhered (10.10 ± 1.72 cells vs 4.4 cells ± 1.01, N = 20; P = 0.005) to the peritoneal vessels in the group treated with Astaxanthin (Fig 4A-4C). These results tempted us to verify potential outside signaling events promoted by ASX. Accordingly, some abdominal aortas of the treated Ldlr^-/-^ mice were used for protein isolation, and phosphorylation of Mitogen-activated protein kinase (MAP) p38 in Western blotting was determined. We observed an enhanced phosphorylation of MAP-kinase p38 in the control group, indicating that known leukocyte activation and adhesion result of MAPK pathway activation, is blocked by Astaxanthin (Fig 4C). These results indicate that Astaxanthin reduces leukocyte adhesion and accumulation via a reduction in the p38 MAPK activation.

### Application of Astaxanthin ameliorates key features of metabolic derangements induced by atherogenic diet in mice

**Fig 5:**
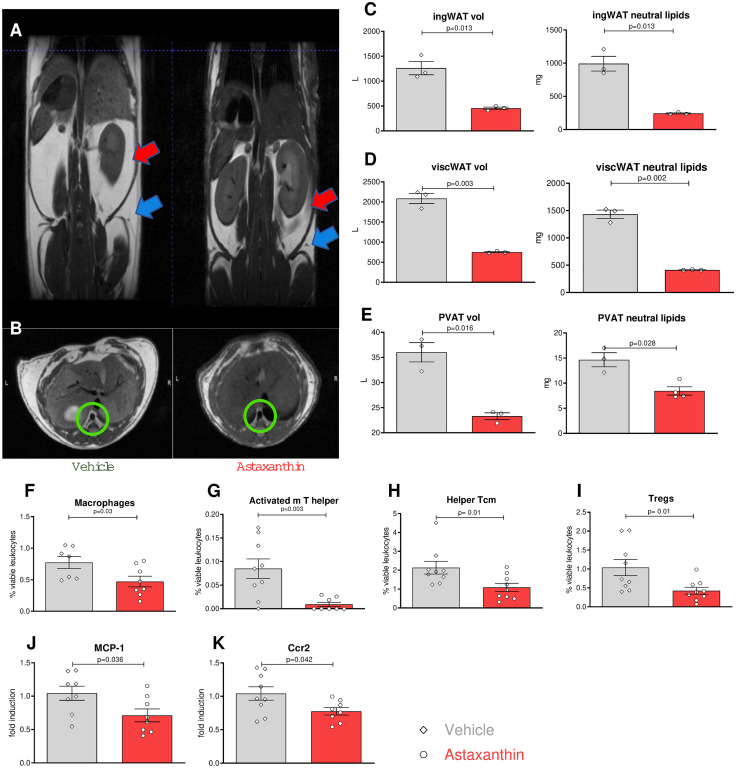
Astaxanthin reduces adipose tissue dysfunction. **(A)** Visceral adipose tissue (red arrow), subcutaneous adipose tissue (blue arrows), and **(B)** perivascular adipose tissue (green circle) were imaged using MRI (white signal). Fat pad volume and neutral lipids in **(C)** inguinal, **(D)** visceral, and **(E)** perivascular compartments. **(F-I)** Leukocyte subsets were identified through flow cytometry of visceral adipose tissue cells after the tissue was digested. Transcripts of MCP-1**(J)** and CCR2 **(K)** measured by qPCR. Error bars represent mean±SEM. Statistical significance was assessed using an unpaired T-test between the indicated groups. N = 9/9 per group (5 males and 4 females).

A diet high in cholesterol and lipids not only induces atherosclerosis but also contributes to the characteristics of metabolic derangements [36,37]. To determine whether ASX impacts the metabolism of Ldlr^−/−^ mice on a high-cholesterol diet (HCD), animals treated with ASX or vehicle control were evaluated for signs of metabolic imbalances. Although body weights throughout the feeding period and the weights of other organs at the conclusion of the study did not vary significantly between the two groups (S3C Fig). To evaluate body fat distribution, mice underwent MRI screening (Fig 5A, 5B), and the volumes of inguinal, visceral, and perivascular adipose tissue were measured. Animals given Astaxanthin demonstrated a significant and consistent decrease in total volume and neutral lipid mass (signal from fatty acid chains) across the three different adipose tissue depots (Fig 5C-5E), indicating that Astaxanthin helps prevent the accumulation of adipose tissue.

A hallmark of adipose tissue inflammation is the infiltration of leukocytes [38]. Therefore, we quantified the number of leukocytes in visceral adipose tissue. Compared to the control group, the group treated with ASX showed a lower number of macrophages (0.77% of viable leukocytes ± 0.09 vs 0.47% of viable leukocytes ± 0.08, N = 18, P = 0.03) and some subtypes of T cells (Fig 5F-5I). Furthermore, MCP-1 and CCR2 mediate monocyte recruitment to white adipose tissue, promoting inflammation and insulin resistance in obesity. Hence their transcript expressions were determined in adipose tissue, and we found a reduction of both in the astaxanthin group (Fig 5J and 5K).

**Fig 6:**
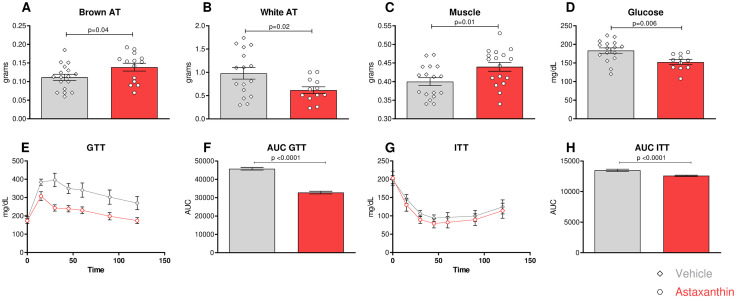
Astaxanthin regulates glucose utilization and insulin sensitivity. **(A-C)** Weight of brown adipose tissue, white adipose tissue, and muscle after 16 weeks of diet and treatment. **(D)**Glucose levels in fasting animals. **(E)** Glucose tolerance testing (GTT), and (G) insulin tolerance testing (ITT). Injections were based on the total body lean tissue weight. **(F, H)** Area under the curve for glucose or insulin tolerance. Error bars represent mean ± SEM. Statistical significance was assessed using an unpaired t-test between indicated groups (A-D) and repeated two-way ANOVA **(E, G)**. N = 16/16 (9 males and 7 females).

Unexpectedly, muscle and brown adipose tissue (BAT) weights were notably greater in the Astaxanthin-treated group (Fig 6A, 6C). Additionally, the ASX group showed a marked reduction in white adipose tissue (WAT) (Fig 6B). One consequence of obesity is the development of insulin resistance and dyslipidemia. However, no differences in plasmatic lipid levels were found (S3 Fig G-I) between the two groups. In line with the reduction of WAT, the group receiving Astaxanthin presented with significantly lower fasting glucose levels (Fig 6D). In accord, the Astaxanthin group showed lower glucose concentrations in blood during an intraperitoneal glucose and insulin tolerance test (Fig 6E-6H). Collectively, our findings identify a protective effect of Astaxanthin against metabolic derangements induced by atherogenic diet.

## Discussion

In this study, we report that astaxanthin reduces atherosclerotic plaque formation and alleviates symptoms characteristic of metabolic syndrome. Our data challenge the traditional view of studying diseases in isolation by presenting results from an atherosclerotic model combined with metabolic data. Hypercholesterolemia has been shown to induce impaired glucose tolerance and insulin sensitivity in Ldlr^−/−^ mice previously due to lower insulin secretion in pancreatic islets, and the insulin secretion defect extended to other fuel secretagogues like l-leucine and 2-ketoisocaproic acid. Cholesterol depletion using methyl-beta-cyclodextrin restored insulin secretion, indicating that the secretion defects were caused by elevated cholesterol [39].

An area of current research is the use of anti-inflammatory agents to battle atherosclerosis and metabolic diseases [40–45]. We previously identified several key inflammatory signal intermediates, such as CD40L, Tumor necrosis factor receptor-associated factors (TRAFs), and danger signaling receptors, as potential targets to regulate the inflammatory balance in this context [23,28,30,33,43,46–49]. It has been shown that Astaxanthin can inhibit specific pro-inflammatory signals in neurological, gastrointestinal, hepatic, and renal diseases [50]. Our data support anti-inflammatory treatment with Astaxanthin, revealing that ASX attenuates the inflammatory response in atherogenesis. While an atherogenic diet is not sufficient in promoting the onset of genuine diet-induced obesity as a model of what we call the metabolic syndrome in humans, it does induce significant metabolic changes, as seen in the glucose- and insulin-tolerance testing we performed. Interestingly, Astaxanthin also blunted those metabolic derangements caused by the atherogenic diet.

In the present study, Astaxanthin not only reduced the development of atherosclerotic plaques in terms of size but also altered their histological characteristics towards features associated with more stability in humans. These findings are consistent with three studies in ApoE knockout mice using natural astaxanthin, Calanus oil (containing astaxanthin and astaxanthin esters), or CX-085, a synthetic drug of the naturally occurring xanthophyll carotenoid astaxanthin, although the postulated mechanisms differ, Ryu et al. and Eilertsen et al. used animal models but did not identify a clear mechanism explaining how astaxanthin influences the development of atherosclerotic plaque. In contrast, Zhang et al. attribute the effect to the CircTPP2/miR-3073b-5p/ABCA1 pathway. However, this mechanism has only been described in macrophages, limiting its generalizability to other cell types [51–53]. Inflammatory processes are orchestrated by a variety of immune cells, among them differentially activated M1 and M2 macrophages [54,55]. Plaques in the astaxanthin group showed a marked reduction in M1 macrophages, known to propagate proatherogenic inflammation. Intimal M1 macrophages are important contributors to atherogenesis; their accumulation progresses during plaque growth and is associated with thrombotic complications [56,57]. The main processes regulating the macrophage content in plaques are adhesion and migration. To better understand the mechanism by which Astaxanthin modulates macrophage content, we tested whether Astaxanthin affects one of these steps with the aid of intravital microscopy. By stimulating mice with TNFα, the endothelium is expected to be activated. Through the interaction of ICAM-1 and VCAM-1, the adhesion of inflammatory cells to the endothelial cell surface is mediated. We identified that a single dose of Astaxanthin before stimulus can significantly reduce inflammatory cell adhesion to the endothelium in mesentery veins *in vivo*. Some studies have reported that the blockade of the MAPK signaling pathway is often related to this inflammatory process [58,59]. Indeed, we could show that p38 phosphorylation is reduced in atherosclerotic aortas from mice treated with astaxanthin.

Several pro-inflammatory cytokines have been found to play a key role in the development of atherosclerosis, for example, TNFα produced by CD4 + T cells and myeloid cells, IL6 that depending on the disease stage, can be either pathogenic or protective, and IL1β produced by myeloid cells, responsible for vascular wall inflammation, due to the upregulation of adhesion molecules by endothelial cells as well macrophage activation [60–64]. Equally, these cytokines have been linked to the onset and progression of adipose tissue inflammation and metabolic syndrome [65–67]. Our experimental data support the idea that astaxanthin is anti-inflammatory, demonstrating inhibition of several of these pro-inflammatory cytokines in our hands, another potential mechanism likely contributing to the effects observed on atherosclerosis and associated metabolic derangements.

Adipose tissue inflammation, with an accumulation of leukocytes and the expression of inflammatory intermediaries that preserve the inflammatory response [68], is a hallmark of obesity. Adipose tissue inflammation results in tissue remodeling and insulin resistance [69]. As anticipated, Astaxanthin alleviated the inflammation of obese visceral adipose tissue, improved metabolism, protected against obesity, and reduced ectopic lipid deposition, possibly by decreasing the number of inflammatory monocytes in the periphery. This, in turn, limited adipose tissue inflammation by reducing the infiltration of macrophages and activated T cells. This decrease in adipose tissue inflammation was not entirely unexpected. Several anti-inflammatory compounds have demonstrated a reduction in pro-inflammatory cytokines, a decrease in macrophage infiltration, and a suppression of adipose tissue inflammation [70].

Other organs, such as the liver, skeletal muscle, brown adipose tissue, and the brain, also contribute to metabolic dysfunction. Improvements in adipose tissue inflammation are usually paired with improvements in other organ functions that influence insulin sensitivity [71]. It has been observed that an increase in brown adipose tissue and muscle positively affects glucose and insulin metabolism and body mass [72,73]. Although the precise mechanism remains unclear, the metabolic benefits related to glucose utilization and insulin sensitivity in the group receiving Astaxanthin have been linked to higher ratios of brown adipose tissue and skeletal muscle, both of which are crucial for improved glucose homeostasis.

In conclusion, we present the finding that Astaxanthin limits atherogenesis and improves metabolism in mice. The most likely underlying mechanisms include attenuated macrophage recruitment to the vascular wall and adipose compartment, reduced inflammatory signaling and cytokine production, and tipping body composition to increased brown adipose tissue and muscle. Our data corroborate the notion that Astaxanthin is a promising pharmacological tool for cardiovascular pathologies. Given the effects observed, the economic attractiveness of Astaxanthin formulations, and the potential broad impact on health and socioeconomics, this hypothesis should be tested rigorously in well-controlled large clinical collectives.

## Supporting information

S1 Fig Granulocytes and T cells found in adipose tissue are unaffected by astaxanthin. Eight-week-old male Ldlr^−/−^ mice were fed a high-cholesterol diet for 16 weeks. Frequencies of (A) granulocytes, (B) helper T cells, and (C) cytotoxic T cells in the stromal vascular fraction isolated from white adipose tissue were quantified using flow cytometry. Leukocyte subpopulations were identified as follows: Granulocytes (CD11b+, GR-1+, CD115−), helper T cells (CD3+, CD4+, CD8−), and cytotoxic T cells (CD3+, CD4−, CD8+), expressed as a percentage of all live CD45+ leukocytes. Statistical significance was assessed using a two-sided, unpaired t-test (A-C). n = 6 mice per group (3 male and 3 female).
(TIF)

S2 Fig The myeloid and lymphocytic subsets in the blood, as well as the plasmatic levels of cytokines, were not altered by Astaxanthin. Male Ldlr^−/−^ mice were fed a high-cholesterol diet for 16 weeks, starting at an age of 8 weeks. Absolute numbers of (A) peripheral granulocytes (CD11b+Gr1+), (B) dendritic cells (CD11b+CD11c+), (C) natural killer cells (CD11b-NK1.1+), (D) B cells type a (CD19+CD5+), (E) B cells type b (CD19+B220+CD5-), (F) T cells (CD19-CD3+), (G) helper T cells (CD3+CD4+CD8-) in the blood were analyzed. Plasma levels of pro-inflammatory cytokines (H) interleukin 6, (I) tumor necrosis factor alpha, and (J) interferon gamma were measured. Levels of anti-inflammatory cytokines (K) interleukin 10 and (L) interleukin 12 were also assessed. Statistical significance was evaluated using a 2-sided, unpaired t-test (A-M). n = 9 (5 male and 4 female) mice per group. Data are presented as mean ± SEM.
(TIF)

S3 Fig Astaxanthin treatment does not affect whole body weight during the feeding period neither the weight of different organs nor plasma lipids. Ldlr^−/−^ mice were fed for 16 weeks with a high-cholesterol diet and divided into two groups, treated with either vehicle or Astaxanthin. (A) Absolute body weight after feeding. (B) Body weight during the feeding period. (C) Weight of the heart, (D) liver, (E) kidney, and (F) spleen. (G) Cholesterol, (H) triglycerides and (I) Non-esterified fatty acids levels in plasma. Statistical significance was assessed using a two-way (repeated-measures) ANOVA (B) and an unpaired, two-sided T-test (A, C-F). n = 16 (9 male and 7 female) mice per group. Data are presented as mean ± SEM.
(TIF)

S1 Table List of antibodies used for flow cytometry (FACS) analysis. This table details all antibodies used for flow cytometry experiments, including antigen targets, fluorochrome conjugates and clones. Antibodies were titrated for optimal signal-to-noise ratio prior to use. All staining was performed according to the manufacturer’s recommendations, and appropriate isotype and fluorescence-minus-one (FMO) controls were included for gating.
(PDF)

S2 Table List of primers used in this study. This table summarizes all primers used for qRT-PCR. The table includes primer abbreviation, protein names and sequences (5′→3′).
(PDF)

S1 Data Raw gel images. (A) Total p38 MAPK. (B) Phospho-p38 MAPK (p-p38). (C) GAPDH (loading control). Uncropped and unprocessed gel images corresponding to the Western blot data shown in the main manuscript. Each panel represents the raw data used for quantification and analysis in the corresponding figure of the main text.
(PDF)

S2 Data Minimal data set. Collection of relevant experimental data: (1) body weight over weeks, (2) intravital data, (3) lab blood count, (4) intraperitoneal glucose tolerance testing, (5) insulin tolerance testing, (6–9) FACS results, (10) Oil-red-O staining, (11) Masson’s trichrome staining, (12) cholesterol, (13) triglycerides, (14) NEFA measurements, (15) qPCR data, (16) inflammatory marker levels, (17) magnetic resonance imaging, (18) Western blot quantification.
(XLSX)
