# Supplementary figures and images for: Astaxanthin limits atherosclerosis and dysmetabolism in mice by attenuating inflammatory cell recruitment and signaling
Source: PLoS One. 2025 Oct 31;20(10):e0334410. doi: 10.1371/journal.pone.0334410 (PMC12578156; doi:10.1371/journal.pone.0334410)

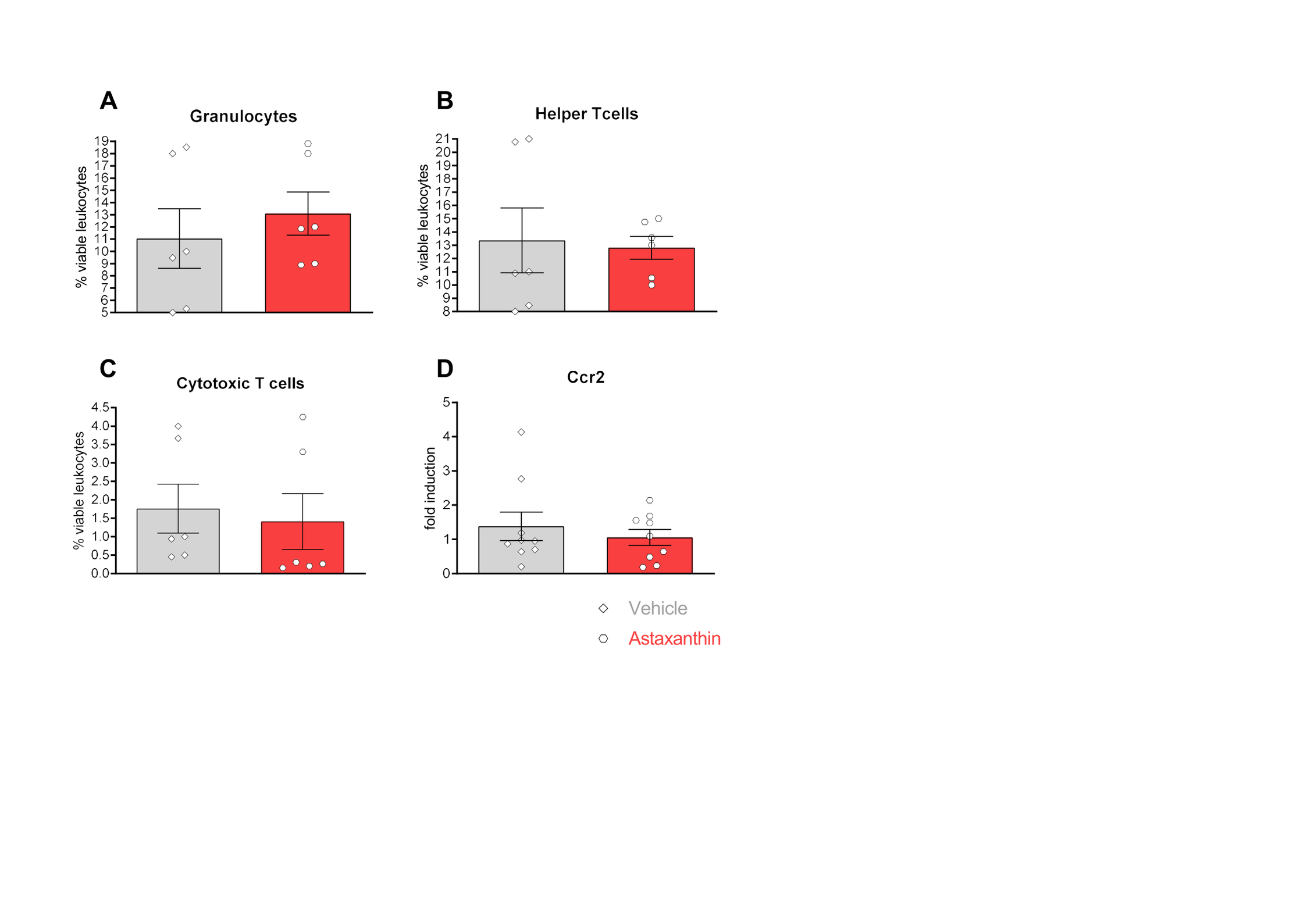

Supplement: S1 Fig — Eight-week-old male Ldlr−/− mice were fed a high-cholesterol diet for 16 weeks. Frequencies of (A) granulocytes, (B) helper T cells, and (C) cytotoxic T cells in the stromal vascular fraction isolated from white adipose tissue were quantified using flow cytometry. Leukocyte subpopulations were identified as follows: Granulocytes (CD11b+, GR-1+, CD115−), helper T cells (CD3+, CD4+, CD8−), and cytotoxic T cells (CD3+, CD4−, CD8+), expressed as a percentage of all live CD45+ leukocytes. Statistical significance was assessed using a two-sided, unpaired t-test (A-C). n = 6 mice per group (3 male and 3 female). (TIF) [file pone.0334410.s001.tif]

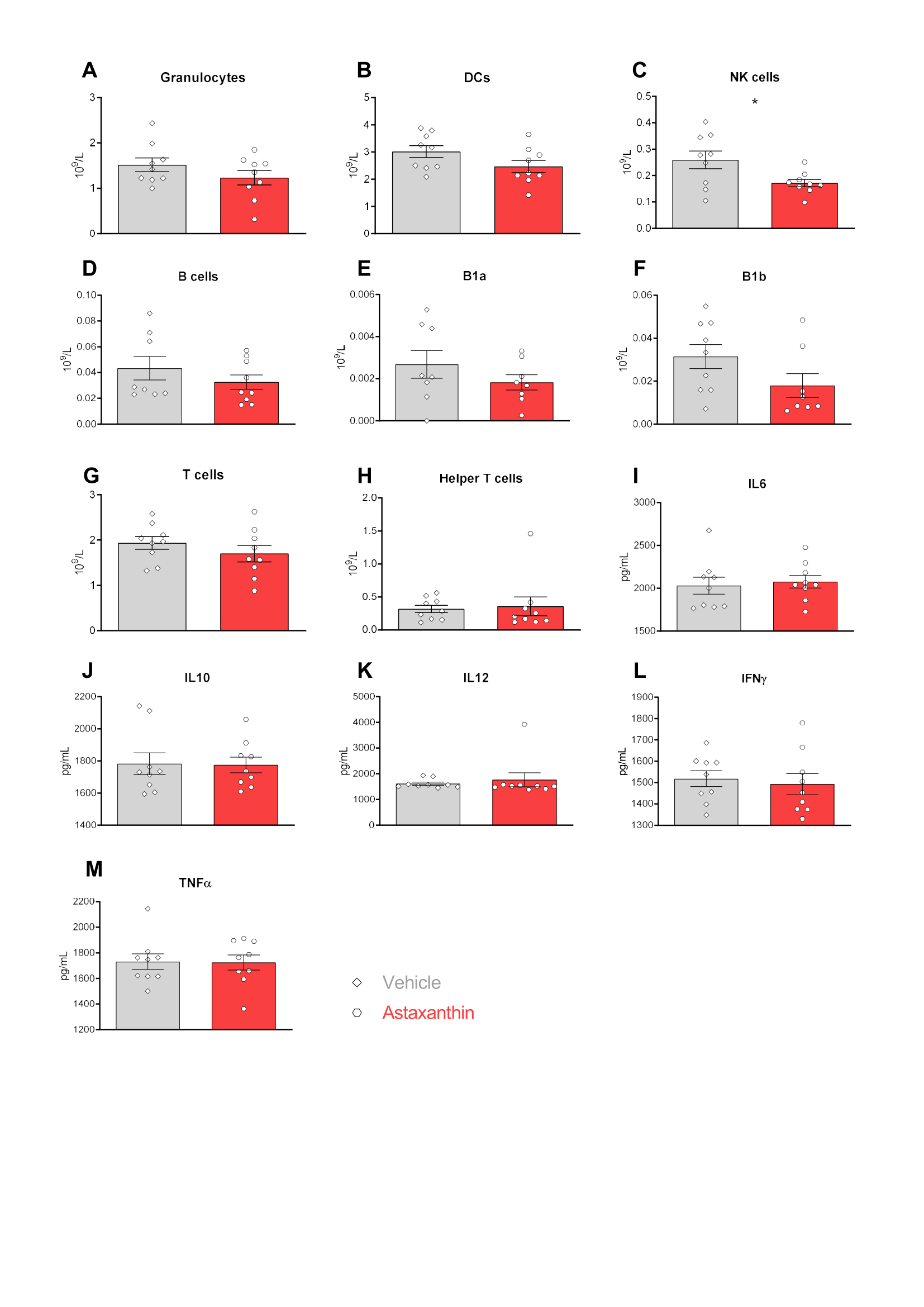

Supplement: S2 Fig — Male Ldlr−/− mice were fed a high-cholesterol diet for 16 weeks, starting at an age of 8 weeks. Absolute numbers of (A) peripheral granulocytes (CD11b+Gr1+), (B) dendritic cells (CD11b+CD11c+), (C) natural killer cells (CD11b-NK1.1+), (D) B cells type a (CD19+CD5+), (E) B cells type b (CD19+B220+CD5-), (F) T cells (CD19-CD3+), (G) helper T cells (CD3+CD4+CD8-) in the blood were analyzed. Plasma levels of pro-inflammatory cytokines (H) interleukin 6, (I) tumor necrosis factor alpha, and (J) interferon gamma were measured. Levels of anti-inflammatory cytokines (K) interleukin 10 and (L) interleukin 12 were also assessed. Statistical significance was evaluated using a 2-sided, unpaired t-test (A-M). n = 9 (5 male and 4 female) mice per group. Data are presented as mean ± SEM. (TIF) [file pone.0334410.s002.tif]

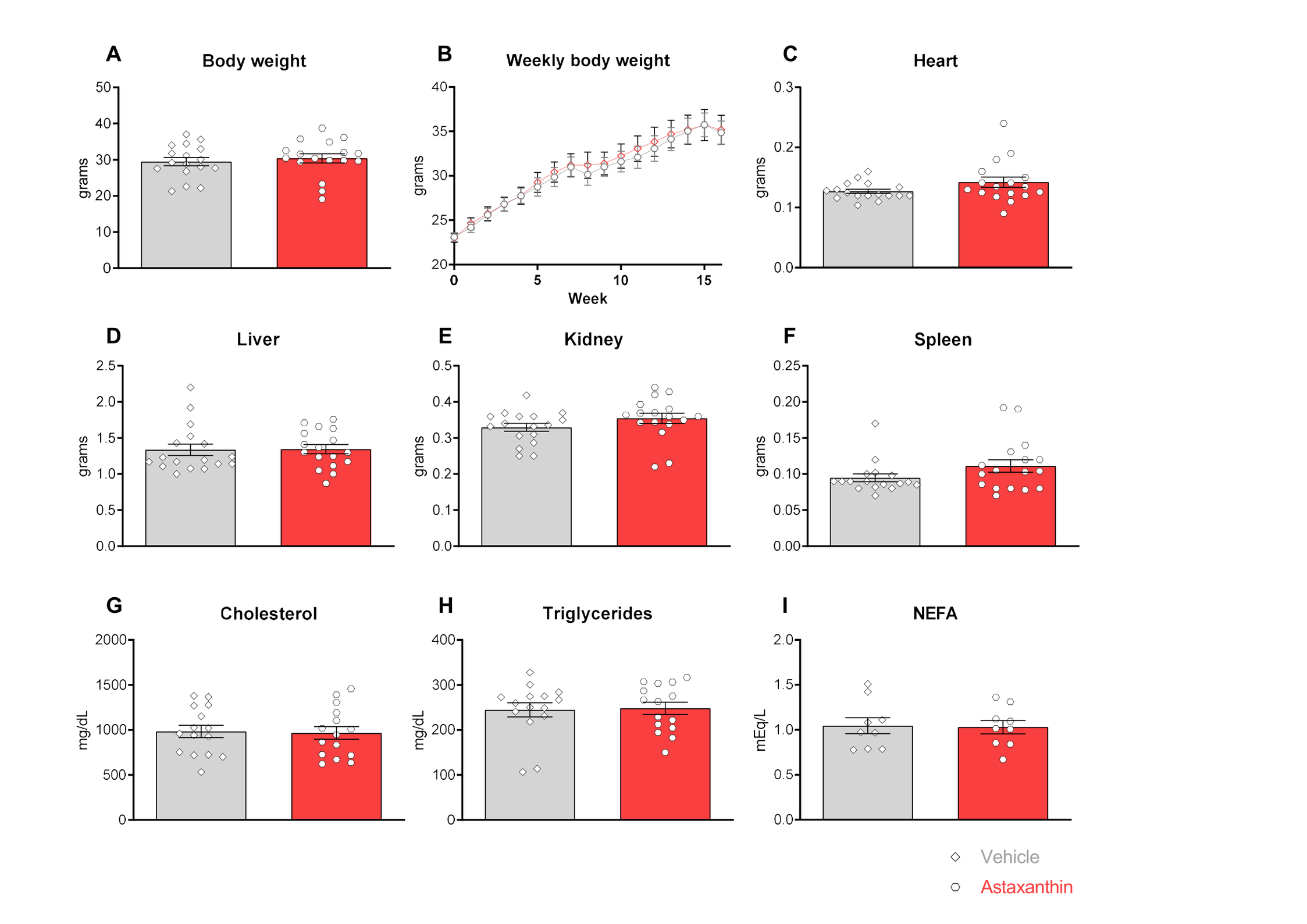

Supplement: S3 Fig — Ldlr−/− mice were fed for 16 weeks with a high-cholesterol diet and divided into two groups, treated with either vehicle or Astaxanthin. (A) Absolute body weight after feeding. (B) Body weight during the feeding period. (C) Weight of the heart, (D) liver, (E) kidney, and (F) spleen. (G) Cholesterol, (H) triglycerides and (I) Non-esterified fatty acids levels in plasma. Statistical significance was assessed using a two-way (repeated-measures) ANOVA (B) and an unpaired, two-sided T-test (A, C-F). n = 16 (9 male and 7 female) mice per group. Data are presented as mean ± SEM. (TIF) [file pone.0334410.s003.tif]

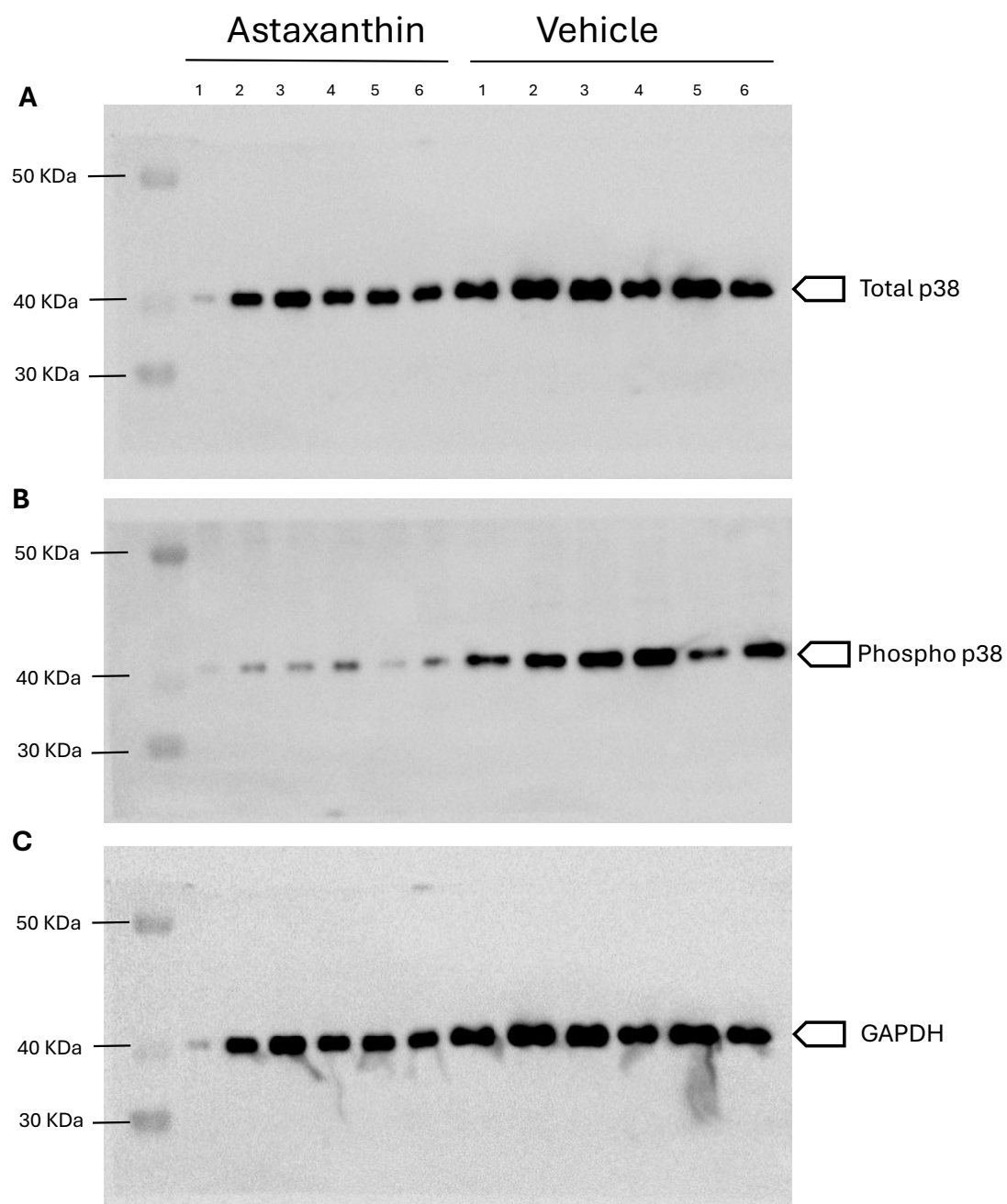

Supplement: S1 Data — (A) Total p38 MAPK. (B) Phospho-p38 MAPK (p-p38). (C) GAPDH (loading control). Uncropped and unprocessed gel images corresponding to the Western blot data shown in the main manuscript. Each panel represents the raw data used for quantification and analysis in the corresponding figure of the main text. (PDF) [file pone.0334410.s006.pdf]
