# Supplementary material for: Astaxanthin limits atherosclerosis and dysmetabolism in mice by attenuating inflammatory cell recruitment and signaling
Source: PLoS One. 2025 Oct 31;20(10):e0334410. doi: 10.1371/journal.pone.0334410 (PMC12578156; doi:10.1371/journal.pone.0334410)
Supplement: S1 Table — This table details all antibodies used for flow cytometry experiments, including antigen targets, fluorochrome conjugates and clones. Antibodies were titrated for optimal signal-to-noise ratio prior to use. All staining was performed according to the manufacturer’s recommendations, and appropriate isotype and fluorescence-minus-one (FMO) controls were included for gating. (PDF) [file pone.0334410.s004.pdf]

S4 Table. FACS antibodies

ANTIBODY	FLUOROCHROME	CLONE
**CD115**	APC	CSF-1R
**CD11B**	PE/Cy7	M1/70
**CD11C**	BV 605	N418
**CD19**	PerCP/Cy5.5	1D3/CD19
**CD3**	APC-Cy7/eFluor 780	17A2
**CD4**	PE-Dazzle	GK1.5
**CD44**	BV650	IM7
**CD45.2**	BUV 395	104
**CD62L**	APC	W18021D
**CD8**	BV 421	53-6.7
**F4/80**	PE	BM8
**FOXP3**	AlexaFluor 647	MF-14
**LY6G**	AlexaFluor 700	1A8
**NK1.1**	BV 650	PK136
**CD5**	PE	53-7.3
**B220**	Violet 421	RA3-6B2
**VIABILITY DYE**	BV 510	"-"
