# Supplementary material for: Astaxanthin limits atherosclerosis and dysmetabolism in mice by attenuating inflammatory cell recruitment and signaling
Source: PLoS One. 2025 Oct 31;20(10):e0334410. doi: 10.1371/journal.pone.0334410 (PMC12578156; doi:10.1371/journal.pone.0334410)
Supplement: S2 Table — This table summarizes all primers used for qRT-PCR. The table includes primer abbreviation, protein names and sequences (5′→3′). (PDF) [file pone.0334410.s005.pdf]

S5 Table. List of Primers

PRIMER	PROTEIN NAME	FORWARD (5'->3')	REVERSE (5'->3')
**m-il6**	Interleukin 6	TTG GTC CTT AGC CAC TCC TTC	TTG GTC CTT AGC CAC TCC TTC
**m-tnf alpha**	tumor necrosis factor alpha	GAG AGT GGT CAG GTT GCC TC	GCA CCT CAG GGA AGA ATC TGG
**m-il1b**	Interleukin 1 beta	CTG CAG CTG GAG AGT GTG G	GGG GAA CTC TGC AGA CTC AA
**m- MCP-1**	Monocyte chemoattracttant protein 1	GCT ACA AGA GGA TCA CCA GCA G	GTC TGG ACC CAT TCC TTC TTG G
**m-Ccr2**	C-C chemokine receptor type 2	GCT GTG TTT GCC TCT CTA CCA G	CAA GTA GAG GCA GGA TCA GGC T
**m-Actb**	beta-actin	CAT TGC TGA CAG GAT GCA GAA GG	TGC TGG AAG GTG GAC AGT GAG G
**m-atpf1**	ATP Synthase Mitochondrial F1 Complex Assembly Factor 1	TGG CGA CAG GCT GGA CTC AG	GCT GCC CGA AGT CTT CTC AGC G
**m- Rplp0**	60S acidic ribosomal protein P0	AAG CGC GTC CTG GCA TTG TCT	CCG CAG GGG CAG CAG TGG T
